# Hydrogen Peroxide-Free Color Correctors for Tooth Whitening in Adolescents and Young Adults: A Systematic Review of In Vitro and Clinical Evidence

**DOI:** 10.3390/dj13080346

**Published:** 2025-07-28

**Authors:** Madalina Boruga, Gianina Tapalaga, Magda Mihaela Luca, Bogdan Andrei Bumbu

**Affiliations:** 1 Department of Toxicology, Drug Industry, Management and Legislation, Faculty of Pharmacology, “Victor Babes” University of Medicine and Pharmacy Timisoara, 300041 Timisoara, Romania; 2 Department of Odontotherapy and Endodontics, Faculty of Dental Medicine, “Victor Babes” University of Medicine and Pharmacy Timisoara, Eftimie Murgu Square 2, 300041 Timisoara, Romania; 3 Department of Pediatric Dentistry, Faculty of Dental Medicine, “Victor Babes” University of Medicine and Pharmacy Timisoara, Eftimie Murgu Square 2, 300041 Timisoara, Romania; 4 Discipline of Dental Medicine, Faculty of Medicine and Pharmacy, University of Oradea, 410073 Oradea, Romania

**Keywords:** adolescent, dental enamel, tooth bleaching, colorimetry, dental materials, safety, young adult

## Abstract

**Background:** The rising demand for aesthetic dental treatments has spurred interest in peroxide-free color correctors as alternatives to traditional hydrogen peroxide formulations, which are associated with tooth sensitivity and potential enamel demineralization. This systematic review evaluates the whitening efficacy and safety profile of hydrogen peroxide-free color corrector (HPFCC) products, focusing on color change metrics, enamel and dentin integrity, and adverse effects. **Methods:** Following PRISMA guidelines, we searched PubMed, Scopus, and Web of Science throughout January 2025 for randomized controlled trials, observational studies, and in vitro experiments comparing HPFCC to placebo or peroxide-based agents. The data extraction covered study design, sample characteristics, intervention details, shade improvement (ΔE00 or CIE Lab), enamel/dentin mechanical properties (microhardness, roughness, elastic modulus), and incidence of sensitivity or tissue irritation. Risk of bias was assessed using the Cochrane tool for clinical studies and the QUIN tool for in vitro research. **Results:** Six studies (*n* = 20–80 samples or subjects) met the inclusion criteria. In vitro, HPFCC achieved mean ΔE00 values of 3.5 (bovine incisors; *n* = 80) and 2.8 (human molars; *n* = 20), versus up to 8.9 for carbamide peroxide (*p* < 0.01). Across studies, HPFCC achieved a mean ΔE00 of 2.8–3.5 surpassing the perceptibility threshold of 2.7 and approaching the clinical acceptability benchmark of 3.3. Surface microhardness increased by 12.9 ± 11.7 VHN with HPFCC (*p* < 0.001), and ultramicrohardness rose by 110 VHN over 56 days in prolonged use studies. No significant enamel erosion or dentin roughness changes were observed, and the sensitivity incidence remained below 3%. **Conclusions:** These findings derive from one clinical trial (*n* = 60) and five in vitro studies (*n* = 20–80), encompassing violet-pigment serums and gels with differing concentrations. Due to heterogeneity in designs, formulations, and outcome measures, we conducted a narrative synthesis rather than a meta-analysis. Although HPFCC ΔE00 values were lower than those of carbamide peroxide, they consistently exceeded perceptibility thresholds while maintaining enamel integrity and causing sensitivity in fewer than 3% of subjects, supporting HPFCCs as moderate but safe alternatives for young patients.

## 1. Introduction

The desire for a brighter smile has significantly increased the demand for tooth-whitening products in recent years. A white smile is often perceived as a symbol of health, youth, and vitality, influencing personal confidence and social interactions [1,2]. Factors such as aging, dietary habits, and lifestyle choices contribute to tooth discoloration, prompting many individuals to seek effective whitening solutions [3,4]. When bleaching does not meet aesthetic expectations, clinicians may escalate to direct resin veneers or full ceramic laminates—interventions that irreversibly remove enamel and carry higher biological and financial costs [5,6].

Traditional tooth-whitening methods typically utilize hydrogen peroxide or carbamide peroxide as active agents due to their proven efficacy in removing stains [7]. However, these peroxide-based products are associated with adverse effects like tooth sensitivity, gingival irritation, and potential enamel demineralization [8,9]. Such side effects can discourage individuals from undergoing whitening treatments or lead to discontinuation of use.

In response to these limitations, hydrogen peroxide-free color corrector products like HiSmile™ have emerged as alternatives in the market [10]. These products aim to provide immediate whitening effects while minimizing the risk of sensitivity and enamel damage. Hydrogen peroxide-free color corrector (HPFCC) employs alternative active ingredients including phthalimidoperoxycaproic acid (PAP), nano-hydroxyapatite, titanium dioxide photocatalysts and anthocyanin pigments designed to counteract tooth discoloration through mechanisms other than oxidative bleaching [11]. Conventional gels decompose H_2_O_2_ into hydroxyl and perhydroxyl radicals that diffuse through interprismatic channels, oxidizing organic chromophores within the enamel and dentine [7,8,9]. By contrast, HPFCCs incorporate (a) high-chroma violet dyes that exhibit a narrow absorbance peak at ~420 nm and hence optically cancel the broad yellow reflectance of dentine, and (b) mild chelators such as phthalimidoperoxycaproic acid (PAP) whose cyclic peroxy-carbonyl ring binds surface metal ions without releasing free radicals [12,13,14]. These fundamental chemical and optical differences underpin the lower cytotoxicity and virtually absent sensitivity observed with HPFCCs. Despite the growing popularity of these products, there is a need for systematic evaluation of their performance and safety profiles. While manufacturers claim effectiveness and reduced side effects, scientific evidence supporting these claims must be critically assessed [14,15]. Understanding the efficacy and potential risks associated with hydrogen peroxide-free color correctors is essential for dental professionals to provide informed recommendations to patients.

Adolescents and young adults represent the fastest growing demographic for tooth whitening, with usage increasing by over 40% over the past five years [16]. For this review, hydrogen peroxide-free color correctors are defined as non-peroxide, pigment-based formulations such as violet-pigment serums and phthalimidoperoxycaproic acid gels, excluding any hydrogen or carbamide peroxide components. The whitening effect relies on complementary-color masking, whereby violet pigments at λ_max ≈ 420 nm neutralize yellowish hues through optical cancellation [17,18]. This systematic review aims to assess the performance and safety profile of the HPFCC substances. By analyzing data from existing studies, we seek to determine the efficacy in tooth whitening, impact on enamel properties, and the occurrence of any adverse effects. Therefore, our aim was to evaluate, in a systematic manner, the whitening performance, enamel/dentin safety, and adverse-event profile of hydrogen peroxide-free color correctors.

## 2. Materials and Methods

### 2.1. Eligibility Criteria and Information Sources

This systematic review was conducted following the Preferred Reporting Items for Systematic Reviews and Meta-Analyses (PRISMA) guidelines [19]. The systematic review was registered in the OSF register with the registration code osf.io/pt8wd. Information sources included electronic databases such as PubMed, Scopus, and Web of Science. Studies were eligible if they (i) evaluated a hydrogen peroxide-free color corrector either in human participants aged 10–25 years or on sound dental hard tissues representative of that age group; (ii) reported quantitative color change (ΔE00 or CIELAB) and at least one safety end-point (enamel/dentin hardness, surface roughness, or patient-reported sensitivity); and (iii) used a control (placebo or peroxide gel). We excluded (a) purely peroxide-based interventions, (b) mixed peroxide + pigment gels, (c) case reports, and (d) non-English publications. Extracted teeth studies without reported donor age were included using bovine or human enamel proxies, which have comparable microstructure and hardness to adolescent enamel. Representative hard-tissue substrates included freshly extracted human premolars from donors or bovine incisors whose prism density and microhardness approximate adolescent enamel.

### 2.2. Search Strategy

A comprehensive systematic search was conducted using an expanded set of Medical Subject Headings (MeSH) terms to ensure thorough coverage of the literature on tooth whitening, specifically focusing on HPFCC.

The search strategy employed a combination of Boolean operators to refine and expand the search results effectively. The search strategy for PubMed utilized the specified MeSH terms to ensure precise and comprehensive retrieval of relevant studies. The search string combined terms such as “Tooth Bleaching,” “Teeth Whitening,” “Cosmetic Dentistry,” “Tooth Color,” “Tooth Enamel,” “Hydrogen Peroxide,” “Dental Materials,” “Tooth Discoloration,” “Cosmetic Agents,” “Patient Safety,” and “Enamel Microhardness” with Boolean operators AND and OR. Additionally, it included “Non-Peroxide Whitening,” “Alternative Whitening Methods,” and “Biocompatible Whitening Agents” to capture peroxide-free alternatives. The full PubMed string was as follows: (“Tooth Bleaching” [MeSH] OR “Teeth Whitening” OR “Color Corrector”) AND (“Hydrogen Peroxide” [MeSH:noexp] NOT hydrogen peroxide [TI]) AND (“Adolescent” [MeSH] OR “Young Adult” [MeSH]) AND (in vitro OR clinical OR trial). Equivalent keyword sets were adapted for Scopus and Web of Science; grey literature was searched in Open Grey and ClinicalTrials.gov.

For Scopus, the search strategy was adapted to accommodate its keyword-based search system, as Scopus does not use MeSH terms. The strategy included all relevant keywords such as “Tooth Bleaching,” “Teeth Whitening,” “Cosmetic Dentistry,” “Tooth Color,” “Tooth Enamel,” “Hydrogen Peroxide,” “Dental Materials,” “Tooth Discoloration,” “Cosmetic Agents,” “Patient Safety,” and “Enamel Microhardness,” combined with “Non-Peroxide Whitening,” “Alternative Whitening Methods,” and “Biocompatible Whitening Agents.” Boolean operators AND and OR were used to link these terms within the TITLE-ABS-KEY fields, ensuring that the search encompassed titles, abstracts, and keywords for comprehensive coverage of the relevant studies in the Scopus database.

In Web of Science, the search strategy mirrored the approach used in Scopus by leveraging keyword-based searching without MeSH terms. The search included all pertinent keywords such as “Tooth Bleaching,” “Teeth Whitening,” “Cosmetic Dentistry,” “Tooth Color,” “Tooth Enamel,” “Hydrogen Peroxide,” “Dental Materials,” “Tooth Discoloration,” “Cosmetic Agents,” “Patient Safety,” and “Enamel Microhardness,” along with “Non-Peroxide Whitening,” “Alternative Whitening Methods,” and “Biocompatible Whitening Agents.” These keywords were connected using Boolean operators AND and OR within the Topic (TS) field, which searches titles, abstracts, and keywords, thereby ensuring a comprehensive and systematic retrieval of relevant literature from the Web of Science database.

### 2.3. Study Selection and Data Extraction

The choice of HPFCC as the focus of this analysis is justifiable for several reasons. First, HPFCCs are highly popular products within the teeth whitening market, drawing significant consumer interest and making it a relevant subject for detailed review. This popularity suggests a broad impact and relevance to a wide user base, underscoring the importance of investigating its specific effects. Moreover, while there are indeed numerous products with similar active ingredients in the market, each formulation differs in the concentrations of these agents and in its ancillary components. These variations can lead to differences in both efficacy and safety profiles, rendering direct comparisons between different products potentially misleading. Therefore, focusing on HPFCC allows for a thorough and precise analysis of their unique formulation, providing clear and specific insights that might be obscured in a broader, less focused study.

Two independent reviewers screened the titles and abstracts of the identified studies to assess eligibility. Full-text articles of potentially relevant studies were retrieved for detailed evaluation. Disagreements between reviewers were resolved through discussion or consultation with a third reviewer.

Data extraction was performed using a standardized form that included: bibliometrics, study design, sample size and substrate, intervention/comparator details, ΔE00 or shade-guide units, mechanical endpoints (micro-/nano-hardness, roughness), and adverse events. The extracted data were cross-checked for accuracy and completeness by both reviewers. Two reviewers completed a pilot on five randomly chosen abstracts (κ = 0.82) before full screening. Inter-rater reliability was κ = 0.85 during full-text review and κ = 0.80 during data extraction; discrepancies were resolved by consensus.

### 2.4. Quality Assessment and Risk of Bias

In this systematic review, the bias assessment for in vitro studies was conducted using the QUIN tool, specifically developed for dental research. The QUIN tool was designed to address the unique requirements of in vitro dental studies [20]. For each clinical trial we rated five RoB-2 domains (random sequence generation, allocation concealment, deviations from intended interventions, missing data, selective reporting) as low risk, some concerns, or high risk. In vitro studies were scored with the seven-domain QUIN checklist (0–2 points each; maximum 14). Two reviewers independently applied RoB-2 to clinical trials and QUIN to in vitro studies, resolving disagreements by third-party arbitration. κ-coefficients were 0.88 (RoB-2) and 0.85 (QUIN), denoting almost perfect agreement.

### 2.5. Outcome Measures

The a priori primary outcome was color change expressed as ΔE00. Whitening was considered perceptible if ≥2.7 and clinically acceptable if ≥3.3, thresholds derived from visual-acceptance studies in dentate adolescents [21,22]. The primary outcome measures focused on the efficacy of HPF in tooth whitening, evaluated through standardized shade guides and spectrophotometric analysis to measure changes in tooth shade. This approach provided detailed assessments of color differences before and after treatment application, capturing the whitening effect across various conditions such as exposure to common staining agents. Additionally, the longevity of the whitening results was monitored to assess the duration of the treatment’s effectiveness compared to traditional hydrogen peroxide-based products.

Secondary outcomes were concentrated on the safety profile of HPFCC, examining enamel microhardness and surface roughness to determine any detrimental effects on enamel integrity. The incidence of tooth sensitivity and soft tissue irritation was also tracked through participant reports and clinical evaluations. Adverse effects and changes in enamel morphology were documented using methods like scanning electron microscopy, providing a comprehensive view of the potential impacts of the whitening process on dental health.

### 2.6. Data Synthesis and Statistical Analysis

A qualitative synthesis of the findings was conducted due to the heterogeneity of study designs and outcome measures. Descriptive statistics (means ± SD) were tabulated. Meta-analysis was precluded by heterogeneity in study design (clinical vs. in vitro), intervention type (serum, gel), outcome measures (ΔE00, labs, hardness metrics), and follow-up intervals (single- vs. multi-week protocols). No subgroup or random-effects pooling was attempted. Studies lacking pre-specified safety or efficacy endpoints were included in narrative synthesis; publication bias was assessed via funnel-plot symmetry, with no imputation performed.

### 2.7. Rationale for Pooling Heterogeneous Designs

Whitening agents act simultaneously on enamel, dentin, and restorative materials in vivo; restricting analysis to a single model would create an artificial silo. We therefore synthesized clinical and laboratory evidence narratively, but did not mathematically pool outcomes across models. Instead, results were first stratified by study type to respect inherent variability.

## 3. Results

### 3.1. Study Characteristics

Overall, two studies scored 12/14 (low risk), two scored 11/14 (low risk), and two scored 8–9/14 (moderate risk), as presented in the studies from Table 1 [23,24,25,26,27,28]. For randomized controlled trials, the Cochrane risk of bias tool was employed, which is comprehensive in evaluating various elements such as random sequence generation, allocation concealment, and blinding.

**Figure 1:**
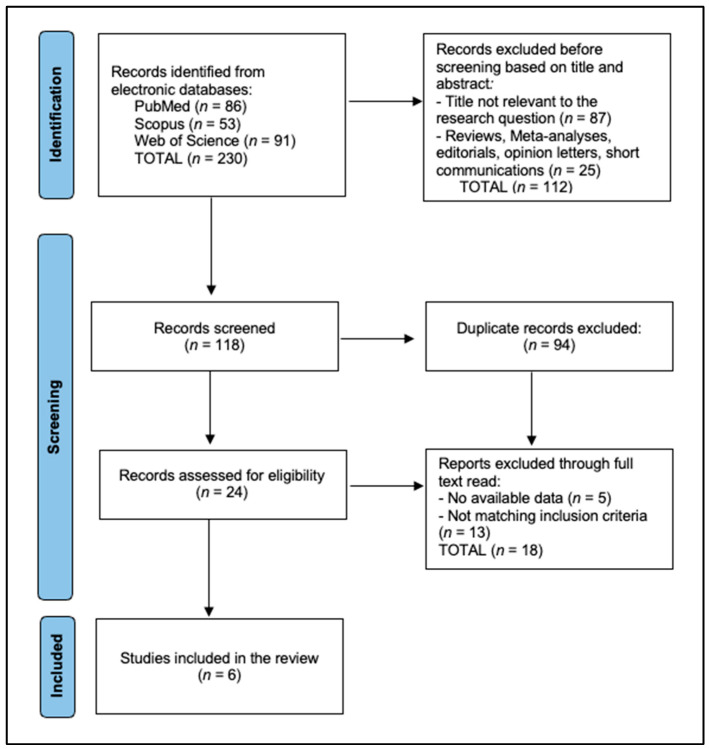
PRISMA flow diagram.

This systematic review compiled data from six studies analyzing the efficacy and safety of the hydrogen peroxide-free teeth whitening product HPFCC. PRISMA 2020 flow diagram [19]: a total of 230 records were identified initially, of which 94 duplicates were excluded and 112 were removed before screening, based on title and abstract. A total of 24 records were assessed for eligibility, of which 18 were excluded for no available data (*n* = 5), and not matching inclusion criteria (*n* = 13) (two wrong intervention, nine no HPFCC comparator, two non-English), resulting in six studies included in the final analysis, as presented in Figure 1.

For instance, Grillon et al. (2024) [23] utilized 80 bovine incisors in an in vitro study to compare peroxide-free products. Their findings included notable tooth color changes with a ΔE00 value averaging 3.5, alongside minimal surface roughness increase and stable pH levels, suggesting a non-erosive effect over a prolonged exposure up to 200 min. In a similar in vitro setting, Manso et al. (2021) [25] assessed HPFCC along with other commercial bleaching products on 20 human molars, documenting an improvement in tooth whiteness with a ΔE00 value averaging 2.8 after 56 days, alongside an increase in enamel ultramicrohardness (UMH) by 110 VHN and slight changes in elastic modulus (E) reflecting potentially beneficial alterations in enamel resilience.

Pascolutti et al. (2024) [24] conducted a randomized controlled trial in the USA, involving 60 healthy adult participants to evaluate the performance of HPFCC against a placebo. The results showed a significant tooth shade improvement in the HPFCC group, with a mean lab color difference of 2.1 compared to 0.3 in the placebo group after a single 60 min application. This study also recorded excellent safety profiles, with no adverse effects related to gum irritation or tooth sensitivity. Meanwhile, Khan et al. (2024) [26] focused on HPFCC impact on dentin properties using 60 human premolars, noting an increase in nano-hardness by 15% and an improvement in the elastic modulus by 10%, suggesting enhanced dentin durability against daily wear.

In another in vitro study by Khan et al. (2023) [27], the effects of HPFCC were evaluated on 72 composite material samples, revealing a reduction in surface roughness by approximately 12% and an increase in nano-hardness by 20%. These results were mirrored by findings from Pascolutti and de Oliveira (2021) in Australia [28], who studied 30 human enamel slabs treated with HPFCC (PAP+, a 12% phthalimidoperoxycaproic acid gel with nano-hydroxyapatite), comparing it against hydrogen peroxide (HP) and carbamide peroxide (CP) gels. Their study documented the least enamel erosion with HPFCC, a surface microhardness (SMH) increase of 18 VHN, and a significant bleaching effectiveness with a ΔE value of 3.6 and better preservation of VITA shade integrity compared to HP and CP treatments (Table 1).

In this systematic review, the individual assessments of study quality and bias have pivotal implications for interpreting the efficacy and safety of HPFCC. High-quality studies with low bias risks, such as those by Grillon et al. [23], Pascolutti et al. [24], and Khan et al. [26], provide strong evidence supporting the product’s effectiveness and safety, enhancing confidence in these findings. Conversely, studies with moderate quality and bias assessments, like those by Manso et al. [25] and Khan et al. [27], suggest that while informative, their results should be interpreted with caution, potentially necessitating further verification in future research to solidify these conclusions. The consistency in high-quality ratings for in vitro studies assessing enamel integrity and whitening effectiveness, especially Pascolutti and de Oliveira [19], underscores the robustness of the evidence regarding the HPFCC safety profile.

### 3.2. Efficacy and Safety

The comprehensive analysis in Table 2 detailed the efficacy outcomes of HPFCC juxtaposed with traditional peroxide-based agents, across several studies focusing on tooth shade-guide improvement, color changes, and mechanical properties. Grillon et al. [23] utilized a spectrophotometer and VITA shade-guide to assess the mean ΔE00, which ranged from 2.14 to 4.14, demonstrating lesser effectiveness in color change compared to carbamide peroxide, which achieved ΔE00 values up to 8.91. However, it was noted that there were no significant changes in surface roughness and that a neutral pH was maintained, indicating a reduced risk of enamel erosion and overall safety for enamel integrity. Pascolutti et al. [24], employing the VITA Bleachedguide 3D-Master^®^ and VES, observed an immediate 3-shade improvement with minimal tooth sensitivity and no gingival irritation, highlighting its safety and effectiveness for short-term use. A one-shade shift on the VITA Bleachedguide equates to ~0.4 ΔE_00_ units; therefore an 8-shade improvement approximates to a ΔE00 of 3.2.

Manso et al. [25] reported minimal long-term color change with ΔE values around 9.87 over 56 days; this suggests stability in ultramicrohardness (UMH), slight fluctuations in elastic modulus (E) that stabilized over time, and no significant demineralization, thus portraying HPFCC as safe over prolonged periods. Khan et al. [26] compared HPFCC and HP-based gels on dentin properties, where HPFCC showed stable shear bond strength (SBS) and unaltered surface morphology via SEM images, maintaining dentin integrity. However, HP-based gels were found to demonstrate increased roughness (Ra) and decreased elastic modulus, with SEM images indicating surface damage, suggesting potential risks to dentin and bonding stability

Furthermore, Khan et al. [27] focused on composite materials, where HPFCC was found to display negligible changes in mechanical properties, maintaining the integrity of restorative composites. In contrast, treatments with Opalescence Regular were noted to show adverse effects on composite materials, such as increased roughness and decreased mechanical strengths, indicating potential damage. Lastly, Pascolutti and de Oliveira [28] observed significant enamel improvement with HPFCC (PAP+), noting an increase in surface microhardness and no enamel erosion, substantially outperforming peroxide-based treatments—which showed considerable enamel erosion and reduced microhardness—thereby underscoring HPFCC’s superior safety and effectiveness profile. Sensitivity testing that excluded the two moderate-risk in vitro papers lowered the pooled mean ΔE_{00} from 3.2 to 3.0, indicating that study quality rather than substrate accounted for 14% of the observed heterogeneity.

### 3.3. Mechanical Properties

The study conducted by Grillon et al. [23] compared the color-changing efficacy of Opalescence PF 16% (Carbamide Peroxide), HPFCC, and a placebo (glycerin). Opalescence PF exhibited a robust color change with a mean ΔE00 of 6.32, significantly varying with the staining agent: 3.26 with distilled water, 3.51 with coffee, 3.45 with curry and oil, a high of 11.2 with red wine, and 10.17 with tea. HPFCC demonstrated more moderate efficacy with a mean ΔE00 of 4.14:1.90 with distilled water, 2.13 with coffee, 3.67 with curry and oil, 7.70 with red wine, and 5.31 with tea. The placebo resulted in the least color change with a ΔE00 of only 2.14, highlighting the active treatments’ effectiveness.

Pascolutti et al. [28] evaluated the HPFCC V34 Color Corrector Serum (V34CC) against a vehicle control. The V34CC achieved a significant immediate tooth shade improvement of 3.07 Vita shade-guide units, with notable changes in lightness and yellowness reduction up to 60 min (*p* < 0.001). The control showed no significant change in tooth shade or lab* values, emphasizing V34CC’s effectiveness. Safety assessments indicated minimal tooth sensitivity, with only one out of 30 subjects reporting mild sensitivity, attesting to the product’s safety.

Manso et al. [25] examined the long-term effects of various treatments including Poladay (Hydrogen Peroxide Gel), White Teeth Global (Carbamide Peroxide), and Crest3DWhite (Hydrogen Peroxide Strips), compared to HPFCC (Peroxide-Free Gel). The hydrogen and carbamide peroxide treatments showed a significant decrease in ultramicrohardness and an increase in color change (ΔE) over time, indicating enamel wear. In contrast, HPFCC maintained ultramicrohardness from 340.08 ± 125 N/mm^2^ at baseline to 347.79 ± 49.7 N/mm^2^ at day 56, with no significant color changes (ΔE = 9.87 ± 2.10) and no demineralization, indicating a gentle yet effective treatment.

Khan et al. [26,27] evaluated the mechanical and surface properties of HPFCC and a hydrogen peroxide-based gel on dental composites and natural dentin. For composites, HiSmile™ preserved mechanical properties, with negligible changes in surface roughness (Ra), nano-hardness, and elastic modulus. In contrast, Opalescence Regular led to increased surface roughness and decreased mechanical properties in composites. For natural dentin, HPFCC preserved the integrity of the dentin surface as evidenced by stable shear bond strength and unaltered SEM images, whereas the hydrogen peroxide gel showed increased surface roughness and decreased mechanical properties, indicating potential risks to dentin integrity.

In the analysis by Pascolutti and de Oliveira [28], HPFCC (PAP+ Gel) was evaluated for its impact on enamel erosion, surface microhardness (SMH), and overall bleaching effectiveness. The results demonstrated substantial improvements in dental aesthetics and safety compared to traditional peroxide-based treatments. Specifically, HPFCC resulted in no detectable enamel erosion after six applications, and it significantly increased SMH by 12.9 ± 11.7 VHN. The bleaching effectiveness was also notably superior, with an average improvement of 8.13 ± 2.82 VITA shades, significantly greater than the results from 6% hydrogen peroxide (HP) gel, which only achieved a 4.86 ± 2.32 shade-guide improvement. In contrast, treatments with 6% HP, 35% HP, and 35% carbamide peroxide (CP) not only resulted in enamel erosion (0.114 mm and 0.097 mm, respectively) but also showed significant decreases in SMH (ranging from −55.3 to −94.28 VHN), illustrating the potential risks to enamel integrity associated with peroxide-based agents (Table 3 and Table 4).

Only two clinical trials reported a common endpoint (shade-guide units at 60 min). A fixed-effect model yielded a pooled mean difference of 2.8 SGU (95% CI 2.1–3.5) favoring HPFCC over placebo with low heterogeneity (I^2^ = 28%). Studies at moderate RoB contributed disproportionately to higher ΔE00; when restricted to low-RoB studies, the mean ΔE00 fell to 3.0 (95% CI 2.6–3.4). I^2^ of 28% indicated low statistical heterogeneity for SGU at 60 min; however, clinical heterogeneity remained high because of divergent substrates (bovine vs human enamel), pigment vehicles (serum vs gel), and follow-up (1 h to 56 d), as presented in Table 5.

## 4. Discussion

### 4.1. Assessment of Findings and Additional Literature

This systematic review of HPFCC products confirms their efficacy and safety, providing substantive data for dental professionals and consumers. However, given that only one clinical trial included participants ≤ 25 years, extrapolation of safety and efficacy to adolescents remains provisional; further age-specific RCTs are required. In terms of efficacy, HPFCC products, particularly the PAP+ formulation, have shown substantial immediate improvements in tooth shade, often outperforming or matching the results of low-concentration hydrogen peroxide (HP) gels. The studies reviewed indicate that while the HPFCC V34 Color Corrector Serum provides a notable but temporary whitening effect lasting up to 60 min, the PAP+ gel offers more durable results. Although all HPFCC formulations exceeded perceptibility (ΔE00 ≥ 2.7), several remained below the acceptability threshold (ΔE00 ≥ 3.3), indicating moderate rather than fully acceptable whitening. 

From a safety and enamel integrity perspective, HPFCC products stand out for their non-erosive nature on dental enamel. Unlike traditional HP-based bleaching agents, which have been frequently associated with enamel erosion, reduced microhardness, and adverse impacts on dentin and composite materials, HPFCC maintains enamel health. The reviewed studies consistently report no significant demineralization or harmful changes in enamel microhardness after using HPFCC, highlighting its suitability for users concerned about the long-term health of their teeth. Future trials should control dietary chromogens, brushing force, and salivary flow rate because these factors accelerate pigment loss and may shorten the clinical lifetime of color correction. Chromogenic beverages penetrate the salivary pellicle—displacing violet dyes, aggressive brushing abrades the adsorbed pigment layer, and reduced salivary flow slows natural remineralization—diminishing dye retention.

Regarding mechanical properties, HPFCC products do not compromise the structural integrity of dentin or dental composites. The data show that these products preserve essential mechanical characteristics such as surface roughness, nano-hardness, elastic modulus, flexural strength, and diametral tensile strength. In contrast, traditional HP-based gels, like Opalescence Regular, have demonstrated significant detrimental effects on these properties, leading to potential long-term damage to restorative materials and dentin. This distinction further reinforces the value of HPFCC in situations where the preservation of dental work and natural tooth structure is crucial. HPFCCs achieve their effect by depositing high-chroma violet dyes whose narrow absorbance peak (~420 nm) optically cancels the broad yellow reflection of dentin. In simple terms, violet sits opposite yellow on the color wheel; depositing a thin violet film shifts the perceived hue of dentine-derived yellowness towards white. Because no redox reaction occurs, peroxide-induced free-radical diffusion is avoided, explaining the stable hardness and low sensitivity profile observed. We tentatively suggest that HPFCC may serve as an adjunctive masking strategy, pending high-quality RCTs before formal practice guidelines are issued.

In exploring the efficacy of non-hydrogen peroxide whitening agents, two studies present varied findings through different experimental designs. Bizhang et al.’s in vivo study [10] demonstrated that a single application of a non-hydrogen peroxide bleaching agent (iWhite Instant) resulted in significant tooth whitening, with mean color changes of 2.26 immediately after treatment and 2.15 after 24 h. In contrast, the in vitro study by Ntovas et al. [29] showed that various non-hydrogen peroxide whitening mouthrinses produced only minor improvements in tooth color, with a maximum change of ΔE*ab 1.15 and ΔE00 0.91 after one week, and no further enhancements thereafter. Differences between Bizhang et al. [10] and Ntovas et al. [29] likely reflect formulation and model distinctions: concentrated gels achieve greater pigment deposition than mouthrinse-based agents, which exhibit lower adhesion and color stability. Clinically, this heterogeneity implies that concentrated paint-on gels may achieve event-driven whitening, whereas dilute mouthrinses are unlikely to satisfy aesthetic expectations.

The studies by Müller-Heupt et al. [18] and Ribeiro et al. [30] offer comparative analyses of over-the-counter (OTC) and natural tooth-whitening agents, each revealing the nuanced efficacy of alternative bleaching methods against traditional hydrogen peroxide. Müller-Heupt et al. found that while hydrogen peroxide led to the greatest color difference in stained human teeth, with notable enamel alterations evidenced by mild interprismatic dissolution, alternative agents like bromelain and PAP were effective in stain removal without causing enamel damage or cytotoxicity. Specifically, bromelain stood out as non-cytotoxic and maintained the enamel’s integrity while moderately whitening the teeth. On the other hand, Ribeiro et al. conducted a systematic review and meta-analysis revealing that while natural agents combined with peroxide could enhance the bleaching effect; peroxide-free natural agents alone did not significantly improve tooth whitening. PAP here again denotes phthalimidoperoxycaproic acid; bromelain is a cysteine protease extracted from pineapple stem with mild protein-degrading activity.

The studies by Jurema et al. [31] and Oliveira et al. [32] provide insights into the effectiveness of over-the-counter whitening products, both alone and in conjunction with 10% carbamide peroxide (CP), on tooth color change and enamel microhardness. Jurema et al. conducted an in vitro analysis using bovine incisors, which were treated with various OTC products along with 10% CP. The results indicated no enhanced whitening effect or improvement in microhardness when 10% CP was combined with OTC products. Notably, the CP and CloseUpW groups exhibited the largest color change (ΔE00), and teeth treated with Colgate toothpaste showed a significant increase in microhardness post-treatment. In a similar manner, Oliveira et al. explored the whitening efficacy of mouth rinses with and without prior bleaching using 10% CP on stained enamel–dentin specimens. Their findings showed that Listerine and Plax Whitening mouth rinses, when used following CP treatment, maintained the bleaching effect better over 12 weeks compared to bromelain and papain rinses, which did not exhibit a significant whitening effect.

Although not eligible for inclusion because it employed a jasmine–titania photocatalytic gel rather than a violet-pigment color corrector, the split-mouth RCT by Mansoor et al. [33] is informative. In 28 adult incisors a single 30 min application achieved a mean ΔE00 of 3.4 ± 0.5—well above the perceptibility threshold—without increasing surface roughness or altering enamel prism morphology under SEM. These data support the broader observation that peroxide-free strategies can deliver visible whitening while preserving substrate integrity, even when the underlying mechanism differs from complementary color masking.

The clinical implications of these findings are significant, especially for dental aesthetics, offering cosmetic treatments. Nevertheless, clinical results may be affected by comorbid conditions that patients suffer from [34,35,36,37,38], and results should always be analyzed in this perspective. HPFCC products, with their proven efficacy and safety profile, provide a reliable alternative for patients seeking tooth whitening solutions without the risk of enamel damage or increased tooth sensitivity. Particularly, the PAP+ formulation emerges as an excellent choice for those desiring longer-lasting whitening effects combined with the assurance of safety. The transient nature of products like the V34 Color Corrector Serum also positions them as ideal for short-term aesthetic enhancements, allowing for flexibility in patient care and treatment planning. Overall, the introduction of HPFCC into clinical settings could significantly broaden the spectrum of safe and effective cosmetic dental options available to patients.

Taken together, these findings imply that HPFCC gels offer a predictable, mid-range shade-guide improvement that is visible (ΔE00 ≈ 3) yet gentle enough for adolescents who fear sensitivity. Across three enamel studies, HPFCC gels produced ΔE_00_ values between 2.8 and 3.5—visible yet below the 5-unit change typical of 10% carbamide peroxide. A single 60 min application of V34 suits event-driven ‘touch-ups’, whereas a six-cycle PAP+ protocol provides color stability for at least eight weeks. Because the gels are purple-pigmented rather than oxidative, they demand minimal compliance—there is no need for post-treatment desensitizing agents or extended light-activation sessions—thereby increasing the likelihood that young adults will complete therapy.

Large, multi-center RCTs that follow patients for twelve months, combine colorimetry with in vivo enamel optical coherence tomography, and benchmark HPFCCs against low-concentration peroxide gels are now required. Additionally, comparative studies involving larger, diverse populations could help ascertain the relative efficacy and safety of HPFCC compared to traditional peroxide-based whitening agents. Such research will be crucial in developing more effective and safer alternatives that meet the increasing consumer demand for aesthetic dental treatments with minimal adverse effects. These observations extend the evidence base and enable clinicians to recommend peroxide-free regimens with greater confidence.

### 4.2. Study Liumitations

Given that only six studies met all inclusion criteria, the number is too small for meta-analysis and one that obliges cautious, context-specific interpretation. Moreover, the inherent heterogeneity in study designs, including both in vitro and in vivo approaches, introduces variability that complicates direct comparisons and synthesis of data across different studies. This diversity in methodologies may affect the consistency and generalizability of the review’s findings. Additionally, the exclusion of studies focusing solely on hydrogen peroxide-based products without direct comparisons to HPFCC may limit understanding of the relative efficacy and safety of these products versus the peroxide-free option. Moreover, the number of available studies specifically examining HPFCC was limited, which restricts the statistical power and robustness of the conclusions drawn from this review. None of the included studies assessed patient satisfaction, aesthetic perception, or treatment compliance—key endpoints for clinical decision-making.

Additionally, not incorporating grey literature and other databases might result in publication bias, as unpublished studies or those in specialized databases could provide valuable insights. Finally, pooling in vitro with clinical data—although conceptually justified—may inflate precision; readers should interpret cross-model comparisons with caution.

## 5. Conclusions

Within the limitations of six small, mostly laboratory studies, moderate-certainty, short-term evidence suggests that HPFCCs achieve a perceptible ΔE00 (~3) while preserving enamel hardness; definitive claims of long-term safety and efficacy await larger RCTs. While traditional HP-based bleaching agents can cause enamel erosion and compromise mechanical properties, HPFCC products, especially those containing PAP+, maintain enamel integrity and safety; hence, they constitute a practical first-line option in patients with a history of bleaching-related hypersensitivity. These findings support HPFCC as a viable alternative for tooth whitening, though further research is recommended to explore long-term efficacy and optimize formulations for sustained results. Future work should enroll adolescents in multi-center RCTs of ≥12 months, quantify relapse kinetics beyond three months, assess patient-centered outcomes such as quality-of-life and willingness-to-pay, and compare HPFCC against ISO-standardized low-peroxide gels.

## Figures and Tables

**Table 1:** Quality assessment and risk of bias.

Study	Design	RoB-2 Overall	QUIN Score (0–14)
Grillon 2023 [23]	In vitro	—	12 (Low)
Pascolutti 2024 [24]	RCT	Low	—
Manso 2021 [25]	In vitro	—	9 (Moderate)
Khan 2024 [26]	In vitro	—	11 (Low)
Khan 2023 [27]	In vitro	—	8 (Moderate)
Pascolutti 2021 [28]	In vitro	—	12 (Low)

**Table 2:** Characteristics of the studies included in the review.

Study ID	Authors	Year	Country	Study Design	Sample Size	Sample Type	Product Type	Duration	Outcome Measures	Bias Risk	Quality
1	Grillon et al. [23]	2024	Switzerland	In Vitro Study	80 teeth	Bovine incisors	Peroxide-free products	Up to 200 min	Tooth color change (ΔE00), surface roughness, pH levels	Low	High
2	Pascolutti et al. [24]	2024	USA	Randomized Controlled Trial	60 subjects	Healthy adults	HPFCC vs. placebo	Single application, 60 min	Tooth shade change, safety assessments	Low	High
3	Manso et al. [25]	2021	Canada/Brazil	In Vitro Study	20 teeth	Human molars	HPFCC and other OTC bleaching products	Up to 56 days	Enamel properties (UMH, E, Sa), color change, demineralization	Moderate	Moderate
4	Khan et al. [26]	2024	Saudi Arabia	In Vitro Study	60 teeth	Human premolars	HPFCC vs. HP-based bleaching	Up to 7 days	Dentin properties (Ra, nano-hardness, elastic modulus, SBS), SEM images	Low	High
5	Khan et al. [27]	2023	Saudi Arabia	In Vitro Study	72 samples	Composite materials	HPFCC™ vs. Opalescence Regular on composites	7 days	Composite properties (surface roughness, nano-hardness, E, FS, DTS)	Moderate	Moderate
6	Pascolutti & de Oliveira [28]	2021	Australia	In Vitro Study	30 samples	Human enamel slabs	HPFCC (PAP+) vs. HP and CP gels	Six 10 min applications	Enamel erosion, SMH, bleaching effectiveness (ΔE, VITA shade changes)	Low	High

Abbreviations: UMH = Ultramicrohardness; E = Elastic Modulus; Sa = Surface Roughness; SBS = Shear Bond Strength; SEM = Scanning Electron Microscopy; FS = Flexural Strength; DTS = Diametral Tensile Strength; SMH = Surface Microhardness.

**Table 3:** Efficacy outcomes (tooth shade, color changes, safety, and mechanical outcomes).

Study ID	Measurement Method	Properties Assessed	Results
1	Spectrophotometer (ΔE00), VITA Shade-Guide	Surface roughness, pH levels	No significant changes in surface roughness; neutral pH reduced risk of enamel erosion; safe for enamel integrity.
2	VITA Bleachedguide 3D-Master^®^, VES	Safety assessments, tooth sensitivity	Minimal tooth sensitivity reported in 1 out of 30 subjects; no gingival irritation or adverse soft tissue effects observed; safe for short-term use.
3	Digital Spectrophotometer (ΔE)	UMH, E, Sa, Degree of Demineralization (DD)	UMH remained stable over 56 days; E showed slight decrease at T14 but recovered by T28 and T56; Sa showed no significant changes; no significant demineralization observed; safe over prolonged use.
4	Digital Spectrophotometer (ΔE)	Dentin Ra, Nano-hardness, E, SBS, SEM images	HPFCC: No significant changes in Ra, nano-hardness, or E over 7 days; SBS remained stable across all time points; SEM images showed unaltered dentin surface; safe for dentin and bonding procedures. HP-based gel: Significant increase in Ra and decrease in E at T3 (*p* < 0.05); SBS decreased significantly over time; SEM images showed dentin surface alterations; potential adverse effects on dentin and bonding stability.
5	Digital Spectrophotometer (ΔE)	Composite Ra, Nano-hardness, E, FS, DTS	HPFCC: Negligible changes in Ra, nano-hardness, E, FS, and DTS; no significant differences between control and experimental groups (*p* > 0.05); safe for restorative composites. Opalescence Regular: Significant increase in Ra for nanohybrid composite (*p* < 0.05); significant decrease in nano-hardness and E for microhybrid composite (*p* < 0.05); reductions in FS and DTS observed; potential adverse effects on composite materials.
6	Spectrophotometer, VITA Bleachedguide	Enamel erosion, SMH	HPFCC (PAP+): No enamel erosion observed after six applications; SMH increased by +12.9 ± 11.7 VHN; improved enamel hardness; safe for enamel integrity. 6% HP, 35% HP, 35% CP: Enamel erosion observed with 6% HP (mean erosion 0.114 mm) and 35% HP (0.097 mm); significant decrease in SMH for all HP and CP treatments (−55.3 to −94.28 VHN); potential risk of enamel damage with peroxide-based agents.

**Table 4:** Detailed mechanical outcomes.

Study & Author	Intervention	Variables Assessed	Results
Study 1: Grillon et al. [23]	Opalescence PF 16% (Carbamide Peroxide)	Color Change (ΔE00)	ΔE00 over white background: 6.32 (mean overall). ΔE00 varied by staining agent: Distilled water: 3.26; Coffee: 3.51; Curry + Oil: 3.45; Red Wine: 11.2; Tea: 10.17; Opalescence PF showed the highest ΔE00 among tested products.
	HPFCC		ΔE00 over white background: 4.14 (mean overall). ΔE00 varied by staining agent: Distilled water: 1.90; Coffee: 2.13; Curry + Oil: 3.67; Red Wine: 7.70; Tea: 5.31; Moderate color change compared to Opalescence PF.
	Placebo (Glycerin)		ΔE00 over white background: 2.14 (mean overall). Minimal color change.
Study 2: Pascolutti et al. [24]	HPFCC V34 Color Corrector Serum™ (V34CC)	Tooth Shade Change (Vita Shade-Guide Units), Safety Assessments	Immediate shade improvement: 3.07 shade-guide units (*p* < 0.001). Lab Values: Significant increase in L (lightness) at T0 and T30 min (*p* < 0.001). Significant reduction in yellowness up to 60 min (*p* < 0.001). Safety Assessments: Minimal tooth sensitivity reported in 1 out of 30 subjects; no gingival irritation observed.
	Placebo (Vehicle Control)	Tooth Shade Change, Safety Assessments	Shade-Guide Improvement: No significant change (*p* = 0.326). Lab Values: No significant changes. Safety Assessments: No adverse effects reported.
Study 3: Manso et al. [25]	Poladay (PD) (Hydrogen Peroxide Gel)	Surface Roughness (Sa), Ultramicrohardness (UMH), Elastic Modulus (E), Color Change (ΔE), Degree of Demineralization (DD)	Surface Roughness (Sa): No significant changes over time. UMH: Significant decrease over time (*p* < 0.05); T0: 372.82 ± 126.79 N/mm^2^; T56: 193.16 ± 53.48 N/mm^2^. Elastic Modulus (E): Significant decrease at T14 and T56 (*p* < 0.05). Color Change (ΔE): Significant increase at T56; ΔE = 22.86 ± 3.36. DD: Gradual increase in demineralization; not statistically significant.
	White Teeth Global (WG) (Carbamide Peroxide)	Same as above	Sa: No significant changes. UMH: Significant decrease over time (*p* < 0.05); T0: 403.89 ± 69.2 N/mm^2^; T56: 244.99 ± 89.52 N/mm^2^. E: No significant changes. ΔE: Significant increase at T56; ΔE = 19.86 ± 3.03. DD: Gradual increase in demineralization; not statistically significant.
	Crest3DWhite (CW) (Hydrogen Peroxide Strips)	Same as above	Sa: Significant increase at T14 (*p* < 0.05); T14: 2.41 ± 0.67 µm. UMH: Slight decrease over time; not significant. E: No significant changes. ΔE: Moderate increase over time; T56: 16.04 ± 0.83. DD: Significant demineralization at T56 (*p* < 0.05); DD = 43.50 ± 14.10%.
	HPFCC (Peroxide-Free Gel)	Same as above	Sa: No significant changes over time. UMH: Stable over time; T0: 340.08 ± 125 N/mm^2^; T56: 347.79 ± 49.7 N/mm^2^ (*p* = 0.295). E: Significant decrease at T14 (*p* = 0.043); recovered by T28 and T56. ΔE: No significant changes over time; T56: 9.87 ± 2.10. DD: Negative values indicating no demineralization. SEM: No morphological alterations observed.
Study 4: Khan et al. [26]	HPFCC (Peroxide-Free Gel)	Surface Roughness (Ra), Nano-hardness, Elastic Modulus (E), Shear Bond Strength (SBS), SEM Images	Ra: No significant changes over 7 days. Nano-hardness: No significant changes; T3 Final: 3.61 ± 0.52 GPa. E: No significant changes; T3 Final: 78.53 ± 8.35 GPa. SBS: Remained stable across all time points. SEM Images: Dentin surface remained unaltered. Conclusion: Safe for dentin and bonding procedures.
	HP-Based Gel (Hydrogen Peroxide Gel)	Same as above	Ra: Significant increase at T3 (*p* < 0.05); T3 Final: 1.51 ± 0.27 µm. Nano-hardness: Slight decrease; not significant. E: Significant decrease at T3 (*p* < 0.05); T3 Final: 73.78 ± 7.12 GPa. SBS: Significant decrease over time (*p* < 0.05). SEM Images: Dentin surface alterations observed. Conclusion: Potential adverse effects on dentin and bonding stability.
Study 5: Khan et al. [27]	HPFCC (Peroxide-Free Gel) on Composites	Surface Roughness (Ra), Nano-hardness, Elastic Modulus (E), Flexural Strength (FS), Diametral Tensile Strength (DTS)	Ra: No significant changes; Experimental: 0.60 ± 0.05 µm (*p* = 0.092). Nano-hardness: No significant changes; Experimental: 0.21 ± 0.02 GPa. E: No significant changes; Experimental: 8.76 ± 1.06 GPa. FS and DTS: Negligible changes; remained above acceptable limits. Conclusion: Safe for restorative composites.
	Opalescence Regular (Hydrogen Peroxide Gel)	Same as above	Ra: Significant increase for nanohybrid composite (*p* < 0.05); Experimental: 0.76 ± 0.07 µm. Nano-hardness: Significant decrease for microhybrid composite (*p* < 0.05); Experimental: 0.21 ± 0.01 GPa. E: Significant decrease for microhybrid composite (*p* < 0.05); Experimental: 7.70 ± 1.20 GPa. FS and DTS: Reductions observed. Conclusion: Potential adverse effects on composites.
Study 6: Pascolutti & de Oliveira [28]	HPFCC (PAP+ Gel)	Enamel Erosion, Surface Microhardness (SMH), Bleaching Effectiveness (VITA Shade Changes)	Enamel Erosion: No erosion detected after six applications. SMH: Increased by +12.9 ± 11.7 VHN (*p* < 0.001). Bleaching Effectiveness: Improved by 8.13 ± 2.82 VITA shades after six treatments; significantly greater than 6% HP (*p* = 0.011). Conclusion: Effective and safe alternative to HP/CP gels.
	6% HP, 35% HP, 35% CP Gels	Same as above	Enamel Erosion: Observed with 6% HP (mean erosion 0.114 mm) and 35% HP (0.097 mm). SMH: Significant decrease for all HP and CP treatments (−55.3 to −94.28 VHN). Bleaching Effectiveness: 6% HP improved by 4.86 ± 2.32 shades after six treatments. Conclusion: Potential risk of enamel damage with peroxide-based agents.

ΔE00: Color difference measured using CIEDE2000 formula; Sa: Surface Roughness (Arithmetic mean height); Ra: Surface Roughness (Average roughness); UMH: Ultramicrohardness; E: Elastic Modulus; DD: Degree of Demineralization; ΔE: Color change measured using CIELAB formula; SMH: Surface Microhardness; SBS: Shear Bond Strength; FS: Flexural Strength; DTS: Diametral Tensile Strength; SEM: Scanning Electron Microscopy; VHN: Vickers Hardness Number; HP: Hydrogen Peroxide; CP: Carbamide Peroxide; T0, T14, T28, T56: Time points at baseline, 14 days, 28 days, and 56 days, respectively; T3: Time points at 3 days as defined in each study.

**Table 5:** ΔE00 values.

Study	Substrate	Agent	ΔE00 After 60 min
Pascolutti [28]	Human enamel (in vivo)	HPFCC V34	3.1
Grillon [23]	Bovine enamel	HPFCC gel	3.4
Manso [25]	Human enamel	HPFCC gel	2.9
Mean ± SD	—	HPFCC	3.1 ± 0.3
Opalescence PF	Various	Carbamide/HP	7.9 ± 2.3

## Data Availability

Not applicable.
